# Local structure in deeply supercooled liquids exhibits growing lengthscales and dynamical correlations

**DOI:** 10.1038/s41467-018-05371-6

**Published:** 2018-08-16

**Authors:** James E. Hallett, Francesco Turci, C. Patrick Royall

**Affiliations:** 1 H.H. Wills Physics Laboratory, Tyndall Avenue, Bristol, BS8 1TL UK; 2 Centre for Nanoscience and Quantum Information, Tyndall Avenue, Bristol, BS8 1FD UK; 3 School of Chemistry, University of Bristol, Cantock’s Close, Bristol, BS8 1TS UK

## Abstract

Glasses are among the most widely used of everyday materials, yet the process by which a liquid’s viscosity increases by 14 decades to become a glass remains unclear, as often contradictory theories provide equally good descriptions of the available data. Knowledge of emergent lengthscales and higher-order structure could help resolve this, but this requires time-resolved measurements of dense particle coordinates—previously only obtained over a limited time interval. Here we present an experimental study of a model colloidal system over a dynamic window significantly larger than previous measurements, revealing structural ordering more strongly linked to dynamics than previously found. Furthermore we find that immobile regions and domains of local structure grow concurrently with density, and that these regions have low configurational entropy. We thus show that local structure plays an important role at deep supercooling, consistent with a thermodynamic interpretation of the glass transition rather than a principally dynamic description.

## Abstract (web summary)

The glass transition remains an unsolved problem due to the scarcity of particle-resolved data over a large dynamic range. Hallett et al. probe an unprecedented time window and show a strong correlation between local structure and slow dynamics in a deeply supercooled liquid of colloids.

## Introduction

Whether the immense increase in viscosity that occurs in glass-forming liquids when they are cooled is related to a true thermodynamic transition at a non-zero (Kauzmann) temperature, or whether it is a kinetic phenomenon associated with structural relaxation times diverging only at zero temperature, remains controversial^1^. A distinctive feature of the glass transition is dynamic heterogeneity, whereby supercooled liquids do not relax uniformly but exhibit fast and slow regions. This may be due to the formation of ‘cooperatively re-arranging regions’ which undergo entropic melting^2^; the hierarchical interactions of mobility excitations^3^; the presence of geometric motifs such as icosahedra^4^ or a transition to a slow-moving phase with high overlap between particle positions^5^. Experimental evidence discriminating between these theoretical approaches would constitute a major step forward in our understanding of the glass transition.

By analogy with conventional phase transitions, one important route to distinguishing these theoretical perspectives lies in identifying relevant lengthscales and their behaviour. The heterogenous nature of viscous liquids allows us to distinguish two main types of lengthscales: dynamical lengthscales, which concern the typical spatial extent of regions with low (or high) mobility (e.g. *ξ*_4_^6,7^, *ξ*_b_^8^ or *ξ*_RG_^9^), and static or structural lengthscales, which quantify the range of (non-crystalline) order (e.g. the point-to-set correlation length *ξ*_PTS_^10,11^ or the extent of domains of particular locally favoured structures (LFS) *ξ*_LFS_^9,12^). Some theories^1,2,4^ anticipate an increasing dynamic lengthscale that is accompanied by a growing structural lengthscale as the temperature is reduced. Detection of this coupled growth would therefore provide significant support for theories that propose a thermodynamic underpinning of the glass transition^1,2,4^, while its absence would favour theories which emphasise the dynamics^3^. Several attempts have been made to find coupled growth between dynamic and structural lengthscales^13,14^, but with mixed results: some find identical scaling between a dynamic correlation length *ξ*_4_ and structural lengthscales in experiment^15^ and computer simulation^16,17^. However, others find that in three dimensions (3D) while *ξ*_4_ increases strongly, structural correlation lengths grow weakly if at all^9,10,13,14,18–20^. Indeed, the lengthscales reported in ref. ^16^ for 3D systems are rather modest, perhaps relating to recent discoveries of significant differences between two and three dimensions for glass-forming systems^21^. Furthermore correlation between LFS and dynamically slow regions has to date been rather limited^22,23^.

Detection of non-crystalline order^24^ such as LFS^4^ requires coordinate data, thus it is generally only possible to directly probe the correlation between structure and dynamics in simulations and particle-resolved experiments. Both these methods provide data over a limited dynamical regime, where the relaxation time of the supercooled liquid is around four orders of magnitude larger than that of the high-temperature liquid^14,25–27^. This corresponds to a regime well described by mode-coupling theory^28–30^, yet at deeper supercooling there is expected to be a crossover from a caged to an ‘activated’ type of dynamics. Therefore between this mode-coupling crossover and the experimental glass transition *T*_g_, we expect new physics not observed at weaker supercooling. In particular, at deep supercooling, relaxation events are expected to be ‘nucleated’ and the nature of the cooperatively relaxing regions is thought to change, in that they are expected to become more compact. Tantalising hints of this behaviour have been gleaned from experiments^11^ and simulation^10^. Pushing past the mode-coupling regime to *T*_g_ in molecular experiments, indirect measurements detect growth of dynamic lengthscales of up to five molecular diameters^13,14,20^. Recent inferences of structural lengthscales suggest a similar (i.e. quite small) value of a few molecular diameters at *T*_g_^20^. In light scattering experiments on colloids, it has also been shown that the temporal evolution of the amounts of locally ordered and slow particles are intimately linked^31^. Yet in order to make progress in directly determining lengthscales and the relationship between local structure and dynamics, we require particle-resolved data closer to *T*_g_ than the mode-coupling regime studied thus far.

Colloidal particles are epitomised by the hard sphere model, for which the phase behaviour as a function of volume fraction (or reduced pressure) is similar to the case of simple molecules as a function of inverse temperature^14,25^. Thus supercooling is analogous to increasing density for colloidal hard spheres and we use the terms interchangeably throughout. To date, particle-resolved studies with colloids of diameter 1.5–3.5 μm^15,25,32,33^ rarely exceed 4 decades of supercooling. Crucially, the mode-coupling crossover is thought to occur after at least 5 decades of supercooling^34^. Since the Brownian time (*τ*_B_ = *σ*^3^*πη*/(8*k*_B_*T*) where *η* is the solvent viscosity and *k*_B_*T* is the thermal energy) taken by dilute colloids of diameter *σ* to diffuse their own radius is typically several seconds, the mode-coupling regime is normally reached on experimental timescales of several days or weeks—beyond the practical limit of most measurements. However, by changing the particle diameter the characteristic timescale of the system can be rescaled. Even decreasing the particle size by little more than a factor of two can increase the dynamic range available by an order of magnitude, sufficient to extend particle-resolved measurements up to and beyond the mode-coupling crossover. Previously, this regime has been accessed with such small colloids using dynamic light scattering^34^ and real-space fluorescent recovery^35^, but in both cases averaged dynamic properties were measured.

Here, we use super-resolution stimulated emission depletion microscopy (STED^36^) to access the detailed structure of a system of small colloids directly in real space with particle resolution. By tracking the particles in space and time, we extend real-space imaging to previously inaccessible timescales. The new dynamical regime we access reveals that the correlation between locally favoured structures and dynamically slow regions is significantly enhanced at deep supercooling. Furthermore, we identify growing static and dynamic lengthscales *ξ*_static_ and *ξ*_slow_, associated with these locally favoured structures and slow-moving regions, respectively. Finally, by measuring the overlap of coordinates in different regions, we obtain evidence consistent with a decrease in the configurational entropy of the system. These results indicate the importance of local structure and its relation to a putative thermodynamic phase transition^2^ in dynamical arrest.

## Results

### Global dynamics

**Fig. 1:**
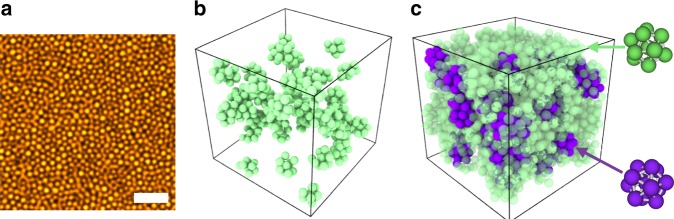
Resolving small colloids in real space. **a** STED nanoscopy image for volume fraction 0.598. Scale bar = 3 μm. **b**, **c** Rendered coordinates of defective icosahedra (green, top right structure) and full icosahedra (purple, bottom right structure) for volume fractions 0.523 (**b**) and 0.598 (**c**), respectively

Figure 1a shows that we are able to resolve particles much smaller than those typically used for particle-resolved studies, and successfully track particle coordinates in 3D to enable structural analysis of the system (Fig. 1b, c). Supplementary Movies 1–4 also show that we may follow the particles over time for a wide range of volume fractions and relaxation times. Moreover, at volume fraction *ϕ* = 0.600, the system is very deeply supercooled—almost seven orders of magnitude slower than the low density liquid. The dynamics at this state point are two orders of magnitude slower than those at the value typically taken for the mode-coupling crossover *ϕ*_MCT_ ≈ 0.58^37^, accessing directly the regime beyond the mode-coupling transition where the mechanism of relaxation is predicted to change^2^.

It has been demonstrated^1,38^ for molecular systems that supercooled liquids continue to relax beyond the mode-coupling crossover, thus we expect to see the equivalent behaviour for this colloidal system. In order to do this we determine the structural relaxation time *τ*_*α*_ from the intermediate scattering function (Methods section) for a range of particle concentrations. We then construct an ‘Angell’ plot of relaxation time, but, rather than *ϕ*, we replot using the Carnahan–Starling equation of state *Z*_cs_ = (1 + *ϕ* + *ϕ*^2^ − *ϕ*^3^)/(1 − *ϕ*)^3^ as a control parameter, following the arguments of Berthier and Witten^39^. This produces similar scaling of the relaxation time compared to thermal systems (where (inverse) temperature is the control parameter)^40^, and gives good agreement with simulations over the range of volume fractions described here^41^.

**Fig. 2:**
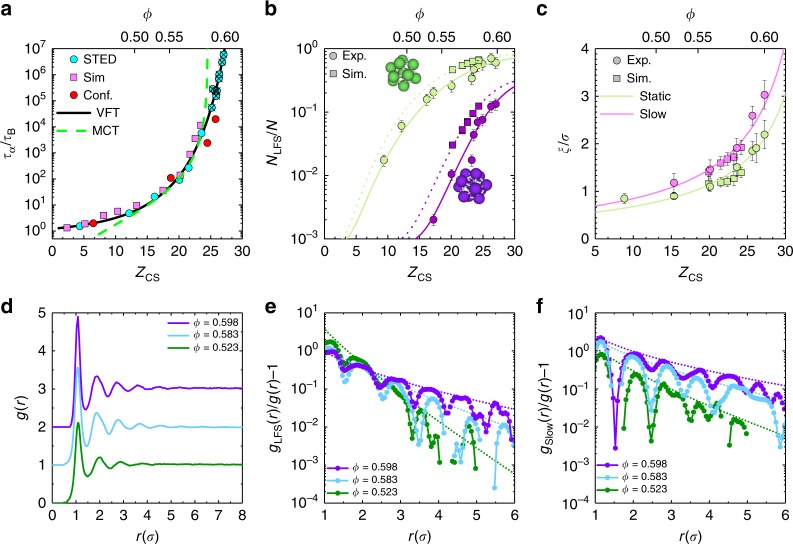
Structure and dynamics at deep supercooling. **a** Structural relaxation time in terms of Brownian time as a function of the reduced pressure *Z* and volume fraction *ϕ* for STED, confocal^44^ and simulations^40^. Key volume fractions are indicated on the top axis. Solid black line is a Vogel–Fulcher–Tammann fit to STED data (see text for details). Dashed green line is a power law fit from mode-coupling theory^1^. Crosses indicate state points equilibrated beyond the avoided mode-coupling transition. **b** Locally favoured structure populations from hard sphere experiments and simulations. Data and structures shown are 10-membered defective icosahedra (green) and 13-membered icosahedra (purple). Solid and dashed lines are a guide to the eye. Error bars correspond to standard deviation. **c** Static and dynamic lengthscales *ξ*_static_ and *ξ*_slow_ obtained for defective icosahedra and slow-moving particles respectively. Solid lines are fitted using Eq. (2). Error bars correspond to standard deviation. **d** Radial distribution function *g*(*r*) for sample state points at weak supercooling, close to the mode-coupling crossover and at deeper supercooling. Curves are offset for clarity. **e** *g*_LFS_(*r*), the spatial correlation of locally favoured structure and **f** *g*_slow_(*r*), the spatial correlation of immobility (Methods section) divided by *g*(*r*) for the same state points as **d**. Dotted lines are fits of the Ornstein–Zernike envelope function Eq. (5)

The resulting Angell plot is shown in Fig. 2a (For convenience we also show the conventional *τ*_*α*_(*ϕ*) form in Supplementary Fig. 4) and two fits are used to describe the increasing structural relaxation time. First, we use a Vogel–Fulcher–Tammann (VFT) form:

1 $$\tau _\alpha \left( \phi \right) = \tau _\infty {\kern 1pt} {\mathrm{exp}}\left[ {\frac{A}{{\left( {\phi _0 - \phi } \right)^\delta }}} \right]$$

where *τ*_∞_ is the relaxation time in a dilute system, *ϕ*_0_ is the point at which the relaxation time would diverge, *A* is a measure of the fragility and *δ* is an exponent typically set to one to recover the conventional VFT form. We see that the VFT fit gives a good agreement with the experimental data throughout the supercooled range. For *δ* = 1, the fit predicts a dynamical divergence at a volume fraction *ϕ*_0_ = 0.616 ± 0.002 (*Z*_0_ = 31.1). Second, we apply a fit of the mode-coupling prediction^1,42^ (Fig. 2a, green line): *τ*_*α*_(*ϕ*) ~ (*ϕ*_MCT_ − *ϕ*)^−*γ*^ where *γ* is ≈2.6 for hard spheres^34^ and *ϕ*_MCT_ is the predicted location of the colloidal glass transition within MCT. Unlike the VFT fit, which produces good agreement at all state points, the mode-coupling theory overestimates the structural relaxation time at high-volume fraction and predicts a dynamical divergence at *ϕ*_MCT_ = 0.590 ± 0.003. Similarly, an extrapolation of the relaxation time for a fixed *γ* of 2.6 at different wavevectors yields *ϕ*_MCT_ = 0.588 ± 0.003^43^ (Supplementary Fig. 5). However, the experiments clearly show a finite relaxation time for *ϕ* > *ϕ*_MCT_. We thus obtain results from direct imaging consistent with those measured using reciprocal space techniques^34^, and demonstrate that our new measurements enable particle-resolved data to be obtained at timescales beyond the mode-coupling crossover.

### Local structure

We now exploit our methodology and investigate local structure at unprecedented degrees of supercooling. In Fig. 1b, c, coordinates are rendered in 3D. Previous work^9,40^ identified a 10-membered structure termed a defective icosahedron as the locally favoured structure for hard spheres. Particles in defective icosahedra are shown in green and particles in regular icosahedra of 13 particles are shown in purple in Fig. 1b, c. At deep supercooling, we see a dramatic increase in the number of particles detected in defective icosahedra, saturating at *N*_LFS_/*N* ~ 0.7 where *N* is the total number of particles detected and *N*_LFS_ is the number of particles found in a LFS. We also see the emergence of a population of particles in full icosahedra, an order of magnitude greater than previously detected in hard sphere experiments^44^. The populations of local structures upon supercooling are shown in Fig. 2b for experiments and simulations. The populations of LFS are slightly lower in experiments than those measured in simulations. We attribute this to small tracking errors (missed particles or positional errors^27^) rather than an uncertainty in *ϕ* between experiments and simulations, due to their close agreement in the Angell plot (Fig. 2a).

That there are more particles in LFS, and that the number of particles in full icosahedra increases by an order of magnitude beyond previous measurements, suggests that some of the less conclusive studies involving LFS at weaker supercooling may not have been able to capture the full link between LFS and the dynamics, due to the limited timescales accessible^16,19,45^.

### Static and dynamic lengthscales

A key means of discriminating between theoretical approaches to the glass transition would be the identification of structural and dynamic lengthscales. To this end, we determine structural and dynamic lengthscales based on 4-point correlation functions following ref. ^6^. We obtain a static lengthscale *ξ*_static_ corresponding to the size of regions rich in locally favoured structures^9,12,46^ and a dynamic lengthscale *ξ*_slow_ corresponding to the immobile or slow-moving regions^6,47^ (Methods section). As expected, the two-point pair distribution function shows little structural change over a range of volume fractions that correspond to nearly seven orders of magnitude of dynamic slowdown (Fig. 2d). However, by plotting the spatial correlation of particles in locally favoured structures (Fig. 2e) and of immobile particles (Fig. 2f) we see a clear separation with increasing density, corresponding to increased spatial correlation between the selected particles. Lengthscales corresponding to the sizes of these correlated regions were then obtained by fitting an Ornstein–Zernike envelope function Eq. (5) to the peaks of these spatial correlations^16^. In Fig. 2c we plot these static and dynamic lengthscales for experiments and simulations and fit them using the following expression, inspired by random first order transition theory^40,48^:

2 $$\xi [Z(\phi )] = \xi _0\left( {\frac{1}{{Z_0 - Z_{{\mathrm{CS}}}(\phi )}}} \right)^{\frac{1}{{3 - \theta }}}.$$

Here *Z*_0_ corresponds to the reduced pressure at which dynamical divergence is predicted from the VFT fit above. We emphasise that, although we reach exceptionally deep levels of supercooling for particle-resolved studies, the state points we equilibrate are still quite far from any true transition, so the lengthscales we identify are modest. Therefore the particular functional form used is a matter of choice, other forms would also fit our data (for example, the power law form taken in ref. ^16^ could also be used). We obtain *ξ*_0_ = 44.7 and *θ* = 2.17 ± 0.12 for the dynamic length and *ξ*_0_ = 22.4 and *θ* = 2.04 ± 0.13 for the static length. The exponent in both cases is the same within errors, so at the level of our analysis the two lengthscales are distinguished only by the prefactor. We see that the structural lengthscale increases significantly, more than doubling in the range we access. Previous efforts to extract a structural lengthscale from defective icosahedra at weaker supercooling only reported a small increase of a factor of ≈1.25^9^. Here the dynamic lengthscale triples, and (within the accuracy of our measurements) grows at a similar rate to *ξ*_RG_, the size of dynamically correlated regions based on a mutual information approach^9^. No evidence of non-monotonicity in either lengthscale^10^ is found.

### Coupling between structure and dynamics

**Fig. 3:**
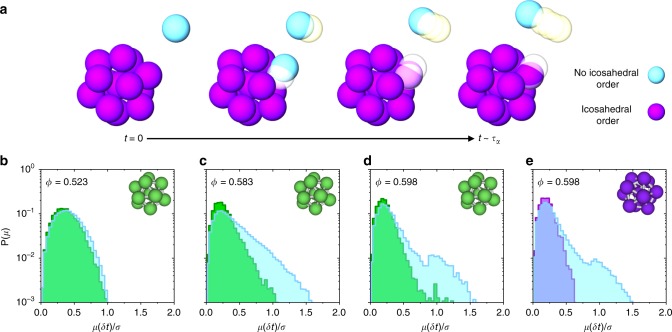
Local structure and dynamic correlations. **a** Schematic of particle displacements for a trajectory that persists in an icosahedral structure and a more mobile, structurally poor trajectory. **b**–**e** Distribution of particle mobility at *δt* ~ 0.5*τ*_*α*_ for defective icosahedra rich (*n*_LFS_ > 0.75, green) and defective icosahedra poor (*n*_LFS_ < 0.25, blue) trajectories, for volume fractions **b** 0.523, **c** 0.583 and **d** 0.598. **e** the same displacements as **d** but for icosahedra rich (purple) and poor (blue) trajectories at volume fraction 0.598

Studies to date have reported some correlation between structure and dynamics^9,16,22^. However, if one expects a causal link between the two, then structural properties should predict dynamical behaviour. Attempts to quantify such predictability have met with only limited success^22,23^. Here we show that a stronger relationship between local structure and dynamics is found at deeper supercooling. In Fig. 3 we quantify the relationship between structure and dynamics by plotting the mobility *μ*_*i*_(*t*) = $\left| {{\bf{r}}_i(t) - {\bf{r}}_i(0)} \right|$ of particles which spend relatively long periods in LFS (>75% of the trajectory) compared to those which spend little time in LFS (<25% of the trajectory), over a time interval ~ 0.5*τ*_*α*_ (Fig. 3a). We express this single-particle trajectory quantity as *n*_LFS_. In this sense, we track the persistence of particles in LFS and correlate it with mobility. (See Supplementary Fig. 6 for LFS persistence over different time intervals). At weak supercooling (*ϕ* = 0.523 (*Z*_cs_ = 15.2), Fig. 3b), there is little distinction between the displacements of particles that persist in local structure and those that do not. This reflects the behaviour observed in Supplementary Movie 1, for which particle motion appears essentially liquid-like and homogeneous. Increasing the volume fraction close to the mode-coupling point (*ϕ* = 0.583 (*Z*_cs_ = 23.8) Fig. 3c), we see a long tail of highly mobile particles which spend little time in defective icosahedra. In other words, fast particles tend not to be found in LFS^9,16,49^. Inspection of Supplementary Movie 2 supports this, where there are spatially distinct fast and slow regions. At *ϕ* = 0.598 (*Z*_cs_ = 26.8), (Fig. 3d), the connection between structure and dynamics strengthens in the mobility distribution, as particles that persist in local structure appear more tightly caged by neighbouring particles than for trajectories at weaker supercooling. For particles with weak persistence in LFS the distribution of displacements is much broader, and the long tail observed for *ϕ* = 0.583 has developed into a secondary peak. This motion is shown in Supplementary Movie 3, where we see significant dynamic heterogeneity. Indeed there are some regions of the sample that undergo relatively persistent cooperative relaxation, while other regions are essentially immobile for the duration of the movie (32 h).

Given the predominance of particles in defective icosahedra for *ϕ* = 0.598 (*Z*_cs_ = 26.8) (*N*_LFS_/*N* ~ 0.6, Fig. 2b), it is likely that some of these particles are relatively less mobile than the others, but detection of defective icosahedra alone does not fully capture this behaviour. Making the comparison of LFS-rich and LFS-poor trajectories for full icosahedra (Fig. 3e) shows a stronger link between structure and dynamics. Certainly, particles in full icosahedra move less than those in defective icosahedra, but the strongest difference compared to weaker supercooling is the distinct second peak at one particle diameter in the tail of fast-moving particles which spend little time in icosahedra, while no particles that persist in icosahedra move so far. This significant population of particles moving around *σ* rather than 2*σ*/3 is consistent with the idea that relaxation may occur through more discrete displacements at these deeper supercoolings, requiring cooperative motion of several particles in a relaxation event. Hints of this can be seen over long timescales (~10^7^*τ*_B_) in Supplementary Movie 3, but this is shown more clearly over intermediate timescales (~2 × 10^4^ *τ*_B_) in Supplementary Movie 4. This shows a relaxation event consisting of cooperative, discrete movements (of around 1 particle diameter) of several particles in an otherwise arrested sample.

### Configurational entropy and local structure

In the previous sections, we have used the notion of LFS as a measure for the increase of structural ordering at high-volume fractions. This assumes that static correlations are captured in LFS domains and that no other mechanisms emerge at high-volume fractions. In order to test this hypothesis we now extend our analysis and consider alternative forms of structural measurement which are order agnostic, that is to say we do not consider any particular local structure.

**Fig. 4:**
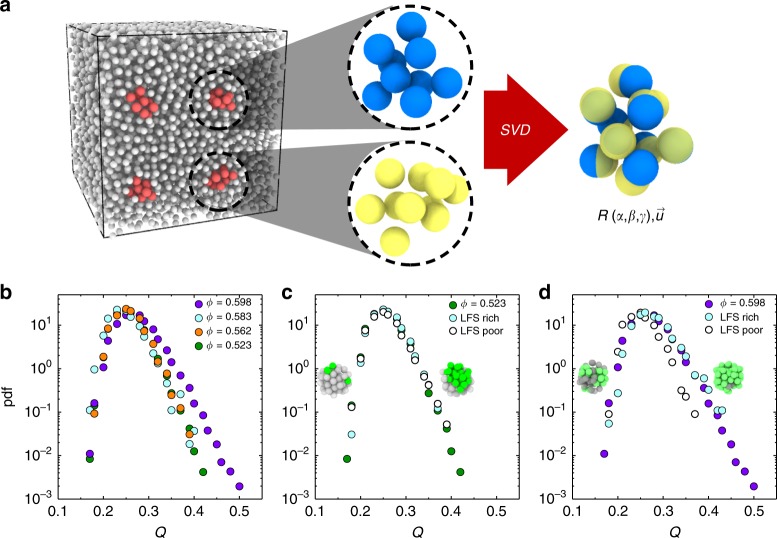
Static overlap fluctuations. **a** Schematic representation of the overlap calculation: isolated clusters of *N*_o_ particles are subsampled from a large configuration; for every pair of clusters, an optimal singular value decomposition (SVD) is found determining the rotation matrix *R*(*α*, *γ*, *β*) and the translation vector $\vec u$ that maximise the overlap. For ease of representation, here *N*_o_ = 10 and *Q* = 0.7. **b** Probability distribution of overlap fluctuations for volume fractions 0.523, 0.562, 0.583 and 0.598. **c**, **d** Overlap fluctuations for all test spheres, and for the most LFS-poor (white) and rich (blue) 25% test spheres, at volume fractions 0.523 (**c** green) and 0.598 (**d** purple). **c**, **d** Samples of LFS-poor (left) and rich (right) test spheres, where green particles are in defective icosahedra and grey particles are not

The first such method is based on the degree of similarity or overlap between different regions of the system^50,51^. High overlap corresponds to very similar configurations (and vice versa). High overlap and thus a low configurational entropy is then expected by thermodynamic-based theories of the glass transition^1,2,5^. We choose to determine the overlap as it has recently been associated with the transition predicted by random first order transition theory^51^. Like the locally favoured structures, overlap is a many-body quantity, and such higher-order measures have been shown to be important in understanding structural quantities in glass-forming systems^14,52,53^. We determine the overlap through subsampling our particle-resolved data into distinct spherical regions which we term test spheres of a fixed number of particles *N*_o_; these are then rotated and translated in order to bring the largest number of centres together, through singular value decomposition; finally the fraction of overlapping particles (i.e. closer than a pre-defined threshold *λ*(*ϕ*)) is measured (Fig. 4a, see Methods for more details). For simulated random configurations, the distribution of overlap values is (trivially) unchanged for all volume fractions. In the experimental system, in the range 0.523 ≤ *ϕ* ≤ 0.583 (15.2 ≤ *Z*_cs_ ≤ 23.8), we see similar overlap distributions as for random configurations at all volume fractions, albeit for higher overlap (Fig. 4b). However, for *ϕ* = 0.598 (*Z*_cs_ = 26.8), the experiments show a clear increase in the occurrence of regions with high overlap. This is an order agnostic indication of a higher degree of structural similarity at deeper supercooling, and a reduction in the number of the sampled possible arrangements, i.e. a lower configurational entropy. We now reverse the argument and ask: is high overlap (amorphous order) related to the LFS considered above? Our answer is positive from the following procedure: we enquire whether particles in the test spheres used in the overlap analysis are in LFS. The overlap distribution was then determined for the most LFS-rich/poor subsets of the overlap population (defined in the Methods). At *ϕ* = 0.523 (Fig. 4c) there is little difference between the total overlap, or the overlaps of structurally rich and poor test spheres. However, at *ϕ* = 0.598 (Fig. 4d) the LFS-poor test spheres clearly do not contribute to the characteristic long tail of overlaps, while the overlap distribution of the LFS-rich test spheres closely tracks the long tail of high overlaps observed for all test spheres. Therefore regions that are rich in icosahedral order are also those with high overlap (and so, low configurational entropy). This demonstrates the compatibility between locally favoured structures and amorphous order.

**Fig. 5:**
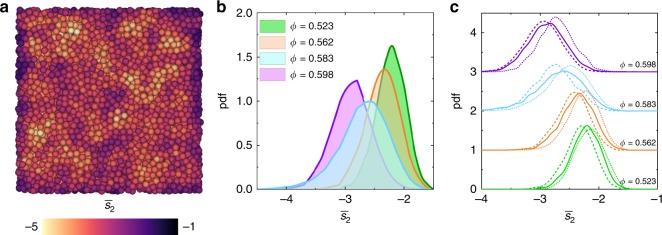
Two-body excess entropy. **a** Spatial distribution of locally averaged two-body excess entropy $\overline s _2$ for volume fraction 0.598. **b** Probability distributions of $\overline s _2$ for a range of volume fractions. **c** Probability distributions of $\overline s _2$ for a range of volume fractions for the whole particle population (solid line), particles identified in defective icosahedra (dashed line) and particles not identified in defective icosahedra (dotted line)

So far we have described local structure and configurational entropy using higher-order properties. However, it is also possible to quantify local entropy from two-point correlations. While there are many different definitions of configurational entropy (in part due to the difficulty in identifying the vibrational contributions), a useful approximation which accounts for about 90% of the configurational entropy can be used for simple liquids, which requires only the pair correlation function as input^53,54^. This forms the basis of the two-body excess entropy contribution to the configurational entropy, or *s*_2_. To more clearly distinguish between different structures the locally averaged form $\overline s _2$, that is to say, the average over nearest neighbours, is preferred^16,54,55^. To assess this, we calculate *s*_2_ and the locally averaged form $\overline s _2$ for a range of state points (see Methods for more information). The distribution of $\overline s _2$ for a deeply supercooled sample is shown in Fig. 5a. This shows correlated regions with high and low local excess entropy distributed throughout the sample. Furthermore, by considering the distribution of $\overline s _2$ at different state points, we see increasing local order (i.e. lower values of $\overline s _2$) with supercooling (Fig. 5b). We now extend our analysis to consider the relationship between $\overline s _2$ and locally favoured structures. By determining the probability distribution function of $\overline s _2$ for particles identified and not identified in LFS, we see that occupancy of LFS consistently predicts particles of lower local excess entropy and vice versa (Fig. 5c). Intriguingly, we also see that for the state point *ϕ* = 0.583 (close to the mode-coupling crossover), the distribution of $\overline s _2$ is relatively broad. A possible explanation for this emerges by considering the role of LFS. The distribution of $\overline s _2$ for the particles in LFS is close to that of the more dense sample and vice versa, indicating that at this state point there are some regions that appear more supercooled and other regions that are more liquid-like, respectively, and selecting for LFS differentiates between the two. We also perform the same analysis for the non-locally averaged *s*_2_ which has much weaker spatial correlations (Supplementary Fig. 7a) and broader distributions (Supplementary Fig. 7b, c) than for $\overline s _2$. This acts to obscure the clear trends shown for $\overline s _2$, consistent with previous observations^54^.

## Discussion

We have used STED microscopy to study the particle-resolved dynamics and structure of glass-forming liquids at unprecedented degrees of supercooling. By obtaining the coordinates of particles much smaller than those imaged previously, our data has effective relaxation times up to three orders of magnitude longer than previous work. Significantly, this deeply supercooled regime passes the mode-coupling crossover, accessing a regime in which new physics is expected^1,2^. We obtained four main results which are explained below.

First, the emergence of a new kind of local structure, the icosahedron, at deep supercooling. This is distinct from, and we believe, more stable than the defective icosahedra identified previously for hard spheres^9^.

Second, at deeper supercooling there is a stronger correlation between dynamics and locally favoured structures. This indicates that earlier work where rather weak correlation was found^9,14,18,19^ may have been influenced by the limited degree of supercooling accessed.

Third, we find a significant increase in the static lengthscale *ξ*_static_ by more than a factor of two and in the dynamic lengthscale *ξ*_slow_ by a factor of three. While this may seem modest, we note that many studies have found it hard even to identify any increase in the structural lengthscale^9,14,18,19^, and that indirect measures of static and dynamic lengthscales at the operational molecular glass transition (seven decades in relaxation time beyond our particle-resolved results) are themselves not much larger than our measurements^20^. We suggest that the results presented here indicate a heightened emphasis of the role of LFS in dynamic arrest at deep supercooling when compared to other structural order parameters.

Finally we present an experimental investigation of the overlap order parameter, related to replica theory^5^. We find evidence of an increase in overlap at deep supercooling. Moreover, we find that regions of high overlap correspond to those rich in LFS, i.e. configurations with low mobility and low configurational entropy. This, combined with the growing amorphous LFS domains, is compatible with the fifth-order dielectric susceptibility measurements on molecular systems reported recently^20^. We also compare this with the local structural entropy and observe similar behaviour as the overlap. That is to say, we see that the distribution of $\overline s _2$ captures the increased local order with volume fraction (Fig. 5). As for the overlap we also see that calculating the same distributions for particles detected in LFS further selects for regions of low configurational entropy.

We now consider the interpretation of our findings in the context of some theoretical approaches. The concurrent growth of the structural and dynamic lengthscales at deeper supercooling points towards the increasing role played by structural correlations in the dynamic slowdown, supporting theoretical approaches which imagine a phase transition related to thermodynamics^1,2,4^. This is also supported by the measurement of the increasing overlap between local regions of the supercooled liquid. However we emphasise that the relatively small lengthscales we obtain mean that our results should be regarded as consistent with some theoretical interpretations rather than anything conclusive. Concerning the connection to theories related to local structure^4^, our data are compatible with geometric frustration, where a frustrated transition to a state of icosahedra is predicted to be suppressed to deep supercooling^12^. We have now accessed such deeper supercooling and find heightened icosahedral ordering. The link between icosahedral ordering and high overlap consolidates the influence of geometric frustration with approaches which emphasise low configurational entropy. Our work thus provides support for thermodynamic (i.e. structural) theories of the glass transition over approaches which prioritise kinetic aspects.

More generally the use of STED microscopy presents several advantages over confocal microscopy for the study of colloidal systems. While the positions of sub-micron systems can be studied using other microscopy methods when confined to a 2D layer^56^ or at lower densities^57^, the key advantage of this approach is that it yields full 3D coordinates at very high densities. This technique provides an enhancement in spatial resolution beyond conventional confocal microscopy, enabling the use of smaller (but otherwise chemically identical) particles. Similar sized particles have previously been studied at comparable densities^34,35^ but crucially those measurements were performed using techniques that do not yield particle positions. Therefore this approach is compatible with the different methods previously applied to the study of dense colloidal hard spheres, but accesses new state points or new information that could not be obtained previously. One difficulty of measurements of this type is that exceptional mechanical and thermal stability is necessary: mechanical drift of the sample of just a few microns (very common during a conventional confocal microscope measurement but negligible compared to the size of the image) can result in the region of interest leaving the imaged volume, prematurely ending the measurement, while thermal fluctuations can result in the laser excitation and depletion spots misaligning, reducing the effectiveness of the STED mechanism. Nevertheless, this new technique holds great promise in the field of colloid and interface science, allowing for systems to be studied in real space that were previously only accessible using scattering or spectroscopic methods.

## Methods

### Sample preparation

Fluorescent dyed-poly methylmethacrylate (PMMA) colloids with a polyhydroxystearic acid comb stabiliser were synthesised using established one-pot dispersion polymerisation methods^58^. To enhance spatial resolution between particles, a non-fluorescent PMMA shell was grown on the fluorescent cores and cross-linked with ethylene glycol dimethacrylate (EGDM) to yield a total radius of 270 nm (polydispersity ~8%, *τ*_B_ = 34 ms). Particle sizes and polydispersity were determined via scanning electron microscopy (SEM) using a JEOL JSM 6330F (Tokyo, Japan). Particles were density and refractive index matched in a solvent mixture of cis-decalin and a saturated solution of tetrabutylammonium bromide salt in cyclohexylbromide (CXB).

Due to the small particle sizes and high-volume fractions used here, re-suspension of dried colloids produced poorly mixed samples. Therefore, to obtain fully dispersed suspensions, PMMA particles were swapped from dodecane to a (non-density matched) mixture of cis-decalin and CXB via repeated centrifugation and replacement of the supernatant with fresh cis-decalin and CXB. Density matching conditions were verified by centrifugation (Eppendorf 5415R temperature controlled centrifuge) for at least 20 min at 13,000 rpm. If the PMMA began to sediment or cream then further cis-decalin or CXB was added until it remained fully suspended after centrifugation. To obtain samples of specific volume fractions, density matched (at room temperature) samples were centrifuged at elevated temperature (to decrease the solvent density relative to the colloids), producing a close packed pellet $(\phi \simeq 0.64)$. The supernatant was removed and the pellet weight was determined. The pellet was then diluted by adding an amount of the supernatant to give a known volume fraction. Samples were then resuspended via vortex and roller mixing. Samples were loaded into cells constructed of three coverslips on a microscope slide, where two of the coverslips acted as a spacer, and sealed using epoxy. Samples were equilibrated while slowly rotating (~1 rpm) in a temperature controlled chamber. The structural relaxation time was monitored for waiting times of up to 100 days until it reached a steady state (Supplementary Fig. 2), at which point the sample was considered to be equilibrated^59^. For example, the state point *ϕ* = 0.598 reached equilibrium after 16 days, corresponding to more than 10*τ*_*α*_. No noticeable crystallisation or sedimentation was detected during this time. Samples were imaged using a Leica SP8 inverted stimulated emission depletion microscope with a ×100 oil immersion lens in STED-3D mode, mounted on an optical table. The optical table was mounted on an acoustically damped five tonne concrete block in a low noise laboratory, to minimise vibrations and instrument drift during measurement. The fluorescent dye was excited using a supercontinuum light source. Depletion was induced using a 660 nm laser. Typically stacks, of volume (10–15 μm)^3^, were recorded with a sampling time between 10 s and 30 min depending on the volume fraction. Measurements were taken at least 20 particle diameters from the cell wall to minimise any structural or dynamic influence of surface layering, which typically persisted for around 5 particle diameters (Supplementary Fig. 3). Images were deconvolved with Huygens Professional version 15.05 prior to analysis (Scientific Volume Imaging, The Netherlands, http://svi.nl).

For samples at low density (*ϕ* ~ 0.50) it was not possible to obtain any dynamic information in the density matching solvent mixture due to the short relaxation times at these state points (*τ*_*α*_ < 500 ms). Therefore in order to obtain data at these volume fractions samples were also prepared in immersion oil (Sigma, *η* = 150 cP), which provided a good refractive index match and slowed down the particles sufficiently to successfully track particle trajectories, at the expense of density matching these suspensions. To minimise the influence of sedimentation during equilibration, these samples were stored slowly rotating prior to measurement. Furthermore, measurements in immersion oil were restricted to shorter times than the self-sedimentation time (~1 h). No noticeable vertical drift or sedimentation was detected during measurement for these samples.

### Particle tracking

Particle tracking algorithms^60^ were used to determine particle coordinates from deconvolved STED microscopy data. The structural relaxation time *τ*_*α*_ was estimated from the intermediate scattering function (ISF), *F*(**k**, *t*) = $\left\langle {\mathop {\sum}\nolimits_{j = 1}^N {\kern 1pt} {\mathrm{exp}}\left[ {i{\bf{k}} \cdot ({\bf{r}}(t + t\prime ) - {\bf{r}}(t\prime ))} \right]} \right\rangle$. This was determine from coordinate data in the case of both experiments and simulations. The lengthscale over which mobility is probed is set by the wavevector *q*, which here was taken to correspond to a particle diameter (*q* ~ 2*πσ*^−1^), close to the main peak in the static structure factor. The long-time tail of the ISF was fitted with a stretched exponential with time constant *τ*_*α*_ and stretching exponent *b*, where typically 0.9 < *b* ≤ 1. Fits to ISFs with a stretched exponential are shown in Supplementary Fig. 1. For some of the samples photobleaching (accelerated beyond conventional confocal microscopy measurements due to the additional contribution of the depletion laser) or sample drift became too significant to accurately track particle positions before the ISF fully decayed. Data for these samples is only reported for the interval where this effect is negligible, as shown in Supplementary Movies 1–4.

In order to verify volume fractions (initially determined by weight), the experimental hard sphere radius was determined by comparison with simulated polydisperse (8%) radial distribution functions of hard spheres. By multiplying the experimental hard sphere volume by the measured number density, a lower bound for the volume fraction of each sample (and their scaling) could be determined. These volume fractions and the scaling of *τ*_*α*_ with number density were referenced against a weakly supercooled fluid state in the regime accessible to computer simulation, with good agreement between their respective *τ*_*α*_ (Supplementary Fig. 4). Rendered images of particle coordinates were produced using Ovito^61^.

We identify local structure from particle coordinate data using the topological cluster classification (TCC)^9,62^. This method defines a bond network to identify arrangements of particles in LFS^62^.

### Lengthscales

Lengthscales were determined from the real space *g*(*r*), subsampled for a particular parameter^9^. For the static lengthscale *ξ*_static_ the density–density correlation was found for particles in LFS:

3 $$g_{{\mathrm{LFS}}}(r) = \frac{1}{{\pi r^2{\mathrm{\Delta }}r\rho \left( {N - 1} \right)}}\mathop {\sum}\limits_{i,j} {\kern 1pt} w_{i,{\mathrm{LFS}}}w_{j,{\mathrm{LFS}}}\delta ( {r - | {\overrightarrow {r_{ij}} } |} )$$

which is the ratio of the ensemble average of particles in LFS at distance *r* (over bin width Δ*r*) to the average number density *ρ*, where *w*_*i*,LFS_ is a binary variable indicating whether particle *i* is in a LFS. A similar approach was used for slow-moving particles to determine the dynamic lengthscale *ξ*_slow_, following^47^:

4 $$g_{{\mathrm{slow}}}(r,\delta t) = \frac{{\mathop {\sum}\limits_{i,j} {\kern 1pt} \mu _{i,{\mathrm{slow}}}\left( {\delta t} \right)\mu _{j,{\mathrm{slow}}}\left( {\delta t} \right)\delta ( {r - | {\overrightarrow {r_{ij}} } |} )}}{{\langle {\mu _{i,{\mathrm{slow}}}\left( {\delta t} \right)} \rangle ^2\pi r^2{\mathrm{\Delta }}r\rho \left( {N - 1} \right)}}$$

where *μ*_*i*,slow_(*t*) is the particle immobility *μ*_max_(*t*) − *μ*_*i*_(*t*) and $\langle {\mu _{i,{\mathrm{slow}}}\left( {{\mathrm{\Delta }}t} \right)} \rangle$ is the average immobility.

The decay of *g*_a_(*r*) was then fitted using an Ornstein–Zernike envelope function^6,9,16^ as follows:

5 $$\frac{{g_a(r)}}{{g(r)}}\sim \frac{1}{r}{\mathrm{exp}}\left( {\frac{{ - r}}{{\xi _a}}} \right)$$

for *a* = LFS or slow, obtaining the structural length *ξ*_static_ and the dynamic length *ξ*_slow_ respectively. We note that these serve as lower bounds for the structural and dynamic lengths due to the size limits of the area that can be imaged by the microscope. For the structural length, this is also due to particles not found or mislocated in the particle tracking analysis, which result in fewer defective icosahedra compared to simulations (Fig. 2b), and thus do not contribute to the lengthscale measurement. For the dynamic length, making the comparison to *χ*_4_, we expect the lengthscale to be maximised at ~*τ*_*α*_^14^. However, this time was not accessible in our most deeply supercooled sample, primarily due to photobleaching at long measurement times, therefore Δ*t* ~ 0.5*τ*_*α*_ was used throughout.

### Simulations

Event-driven molecular dynamics (MD) simulations were carried out with the DynamO package^63^. An equimolar five component mixture of hard spheres of polydispersity 8% (diameters 0.888*σ*, 0.95733*σ*, *σ*, 1.04267*σ* and 1.112*σ*) was used, with a system size of *N* = 1372. At least 3000 configurations were analysed for each state point. Configurations were equilibrated for at least 30*τ*_*α*_ and sampled for at least a further 30*τ*_*α*_, where *τ*_*α*_ was determined by fitting a stretched exponential to the ISF at the main peak of the static structure factor, as for the particle tracking experiments.

### Overlap fluctuations

In order to measure the overlap between distinct regions of the sampled configurations we proceed via subsampling. We select *N*_centres_ equally spaced centres and identify the *N*_o_ = 64 closest particles to the centre in order to obtain *N*_centres_ approximately spherical distinct regions. For every pair of test spheres S_1_, S_2_ we determine the degree of similarity between them performing singular value decomposition. This provides the rotation and translation matrices that maximise the following definition of the overlap:

6 $$Q_{{\rm{S}}_1,{\rm{S}}_2} = \frac{1}{{N_o}}\mathop {\sum}\limits_{i = 1}^{N_o} {\kern 1pt} {\mathrm{\Theta }}( {\lambda - | {r_i^{{\rm{S}}_1} - r_1^{{\rm{S}}_2}} |} ) \vee {\mathrm{\Theta }}( {\lambda - | {r_i^{{\rm{S}}_1} - r_2^{{\rm{S}}_2}} |} ) \vee \ldots \vee {\mathrm{\Theta }}( {\lambda - | {r_i^{{\rm{S}}_1} - r_N^{{\rm{S}}_2}} |} )$$

where Θ is the Heaviside function, *∨* indicates the logical OR operation and *λ* is a cutoff length set to a value that scales as $\lambda = \frac{1}{3}\root {3} \of {{1{\mathrm{/}}\rho }}$, with *ρ* being the number density. This threshold was chosen to be tolerant towards *β* relaxation within otherwise unchanged configurations and corresponded to ~0.4*σ* for all samples analysed. All the possible combinations between the *N*_centres_ spheres are considered in order to obtain a sample of typically *N*_*Q*_ ≈ 10^5^ values for the overlap from which the histograms of Fig. 4 are computed. To determine the overlap distribution of the icosahedrally rich and poor subsets, the product of the fraction of LFS in the test spheres used to calculate the overlap was determined for each overlap value. The overlap distribution was then determined for the overlap values within the highest and lowest 10% of LFS products. This methodology is related to the notion of repeated patches, as previously explored in 2-dimensions^64^. For the overlap measurement of random configurations, the volume fraction controls the number density of particles and the cutoff length *λ*.

### Two-body excess entropy

The two-body excess entropy^16,55^ is given by:

7 $$s_2 = - 2\pi \rho k_{\rm{B}}{\int}_{0}^\infty \left[ {g(r){\mathrm{log}}(g(r)) - g(r) + 1} \right]r^2{\rm{d}}r$$

This can be also be calculated for each particle *i* in the system:

8 $$s_2^i = - 2\pi \rho k_{\rm{B}}{\int}_{0}^\infty \left[ {g_{\rm{m}}^i(r){\mathrm{log}}\left( {g_{\rm{m}}^i(r)} \right) - g_{\rm{m}}^i(r) + 1} \right]r^2{\rm{d}}r$$

where $g_{\rm{m}}^i(r)$ is the radial distribution function for particle *i*. To obtain a continous differentiable order parameter for each particle, we follow^54^, by calculating a ‘mollified’ pair distribution function:

9 $$g_m^i(r) = \frac{1}{{4\pi \rho r^2}}\mathop {\sum}\limits_j \frac{1}{{\left( {2\pi s^2} \right)^{1/2}}}e^{ - \left( {r - r_{ij}} \right)^2/(2s^2)}$$

where *s* is a broadening parameter, *j* are the neighbours of particle *i* and *r*_*ij*_ is their separation. The locally averaged parameter $\overline s _2$ is then determined by averaging the contributions of particle *i* and its nearest neighbours.

### Data availability

The raw data that support the findings of this study are available from the corresponding author upon reasonable request.

## Electronic supplementary material

Supplementary Information

Description of Additional Supplementary Files

Supplementary Movie 1

Supplementary Movie 2

Supplementary Movie 3

Supplementary Movie 4
