# Supplementary material for: Local structure in deeply supercooled liquids exhibits growing lengthscales and dynamical correlations
Source: Nat Commun. 2018 Aug 16;9:3272. doi: 10.1038/s41467-018-05371-6 (PMC6095888; doi:10.1038/s41467-018-05371-6)
Supplement: Supplementary file 1 — Supplementary Information [file 41467_2018_5371_MOESM1_ESM.pdf]

Supplementary Information

Local structure in deeply supercooled liquids exhibits growing lengthscales and dynamical correlations

Hallett et al.

I. SUPPLEMENTARY FIGURES

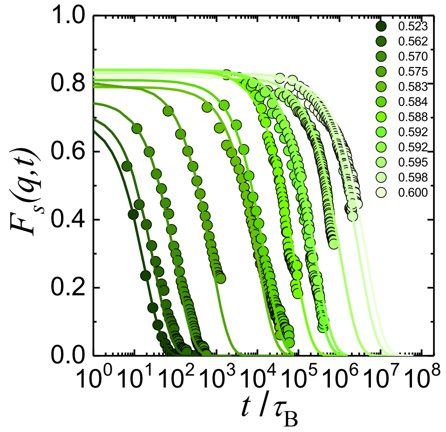

Supplementary Figure 1: **Intermediate scattering functions.** Intermediate scattering function for PMMA particles in immersion oil (at low volume fractions) or CHB−cis decalin index matching mixture for a range of volume fractions. Solid lines are stretched exponential fits.

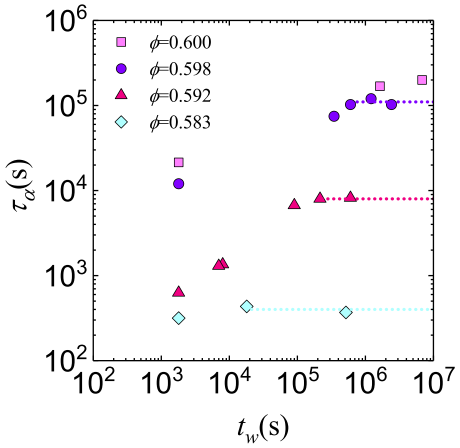

Supplementary Figure 2: **Aging and relaxation.** Age dependence of the alpha relaxation time τ_α for a range of volume fractions. The first measurement was taken approximately 30 minutes after sealing the cell, to allow for the epoxy to cure and to load the sample into the microscope. Horizontal dotted lines correspond to equilibrated alpha relaxation times.

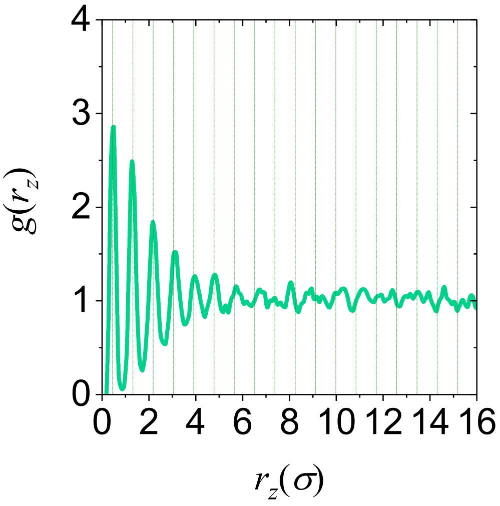

Supplementary Figure 3: **Surface layering.** Surface density distribution for volume fraction $\phi = 0.592$. Distances taken approximately relative to the coverslip. Vertical lines indicate separations expected for hexagonal packing.

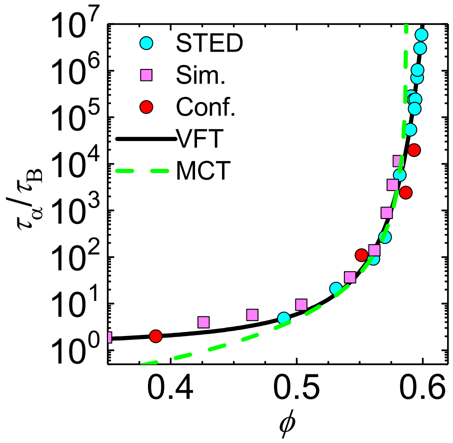

Supplementary Figure 4: **Aging and volume fraction.** Angell plot as a function of volume fraction ϕ for PMMA particles in immersion oil or CHB−cis decalin index matching mixture for a range of volume fractions.

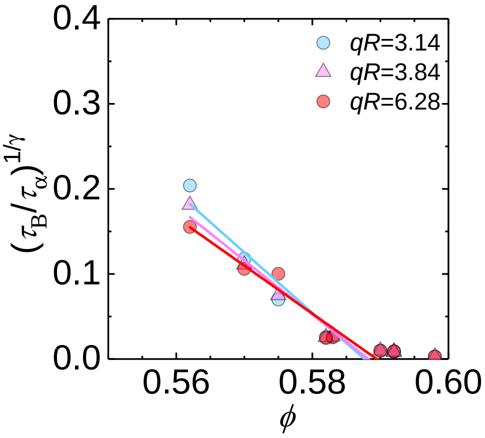

Supplementary Figure 5: **Mode coupling crossover.** Inverse dimensionless relaxation time τ_B/τ_α raised to the inverse of the critical MCT exponent γ as a function of volume fraction for γ=2.6 for wavevectors qR=3.14, qR=3.84 and qR=6.28. Here R is the particle radius. Lines are fits to the intermediate volume fraction region and can be extrapolated to ϕ_{MCT}.

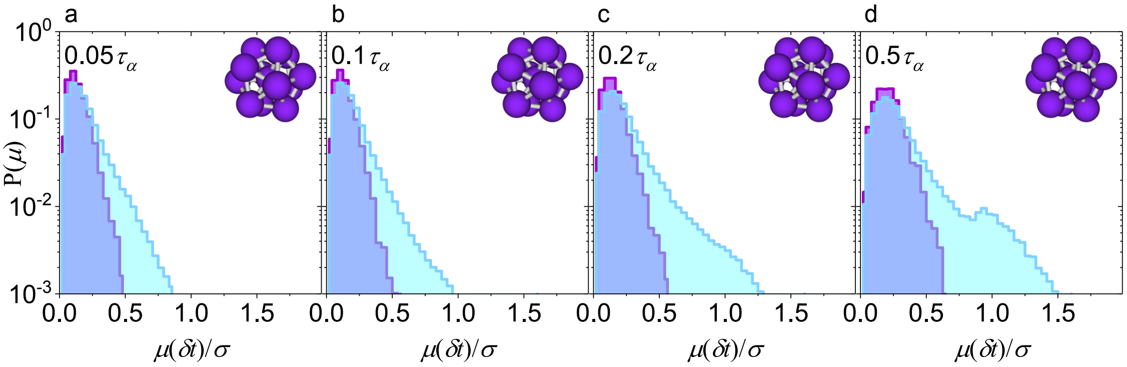

Supplementary Figure 6: **Local structure and trajectory length.** Probability of particle displacement distance at $\phi = 0.598$ for icosahedra rich ($n_{\mathrm{LFS}} > 0.75$, pink) and icosahedra poor ($n_{\mathrm{LFS}} < 0.25$, blue) trajectories, for approximate δt (**a**) $0.05\tau_\alpha$, (**b**) $0.1\tau_\alpha$,(**c**) $0.2\tau_\alpha$ and (**d**) $0.5\tau_\alpha$.

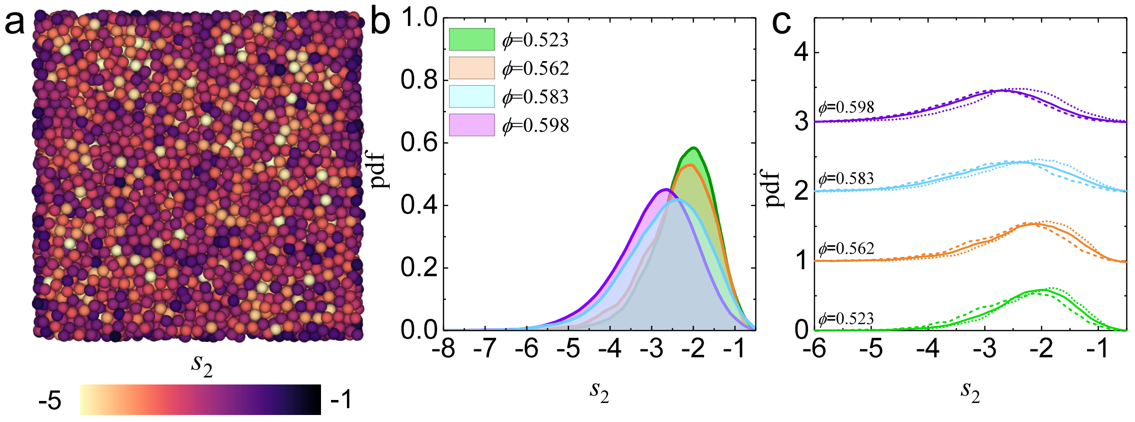

Supplementary Figure 7: **Excess entropy.** **a**, Spatial distribution of the two body excess entropy s_2 for volume fraction 0.598. **b**, Probability distributions of s_2 for a range of volume fractions. **c**, Probability distributions of s_2 for a range of volume fractions for the whole particle population (solid line), particles identified in defective icosahedra (dashed line) and particles not identified in defective icosahedra (dotted line).
