# Supplementary material for: Local structure in deeply supercooled liquids exhibits growing lengthscales and dynamical correlations
Source: Nat Commun. 2018 Aug 16;9:3272. doi: 10.1038/s41467-018-05371-6 (PMC6095888; doi:10.1038/s41467-018-05371-6)
Supplement: Supplementary file 2 — Description of Additional Supplementary Files [file 41467_2018_5371_MOESM2_ESM.pdf]

Description of Additional Supplementary Files

File Name: Supplementary Movie 1
Description: Microscopy video of PMMA particles in immersion oil at volume fraction 0.523, showing liquid-like particle motion. Top and bottom timestamps correspond to Brownian time and seconds respectively. Video duration 23 minutes.

File Name: Supplementary Movie 2
Description: Microscopy video of PMMA particles in cis-decalin and cyclohexyl bromide solvent mixture at volume fraction 0.583, showing the emergence of slow dynamics. Top and bottom timestamps correspond to Brownian time and seconds respectively. Video duration 21 minutes.

File Name: Supplementary Movie 3
Description: Microscopy video of PMMA particles in cis-decalin and cyclohexyl bromide solvent mixture at volume fraction 0.598, capturing long-time relaxations. Cooperatively rearranging regions can be observed, particularly to the right and left of the video centre. Video duration 32 hours. Top and bottom timestamps correspond to Brownian time and seconds respectively.

File Name: Supplementary Movie 4
Description: Microscopy video of PMMA particles in cis-decalin and cyclohexyl bromide solvent mixture at volume fraction 0.598. Discrete, cooperative relaxations are followed, particularly in the central quadrant. Top and bottom timestamps correspond to Brownian time and seconds respectively. Video duration 15 minutes.
